# Incidence of Uterine Cesarean Scar Niche After Cesarean Delivery and Assessment of Its Risk Factors

**DOI:** 10.3390/medicina61091621

**Published:** 2025-09-08

**Authors:** Ahmed Khedr Khalifa, Ahmed Adel Yasseen Abdel Moteleb, Marwa O. Elgendy, Ahmed Abdel Khalek Taha, Eman A. Salem, Ahmed R. N. Ibrahim, Sara Abdallah Mohamed Salem, Eman Zein Elabein Farid, Waleed Mohammed Elamin Khaled

**Affiliations:** 1 Obstetrics & Gynecology Department, Faculty of Medicine, Beni-Suef University, Beni Suef 62511, Egypt; ahmedkhedrkh@gmail.com (A.K.K.); ahmed.adel@med.bsu.edu.eg (A.A.Y.A.M.); ahmedabdulkhaliq@med.bsu.edu.eg (A.A.K.T.); sara.abdalla@med.bsu.edu.eg (S.A.M.S.); eman.zain@gmail.com (E.Z.E.F.); waliedelamin123@gmail.com (W.M.E.K.); 2 Department of Clinical Pharmacy, Beni-Suef University Hospitals, Faculty of Medicine, Beni-Suef University, Beni Suef 62511, Egypt; 3 Department of Clinical Pharmacy, Faculty of Pharmacy, Nahda University (NUB), Beni Suef 62764, Egypt; 4 General Surgery Department, Faculty of Medicine, Beni-Suef University, Beni Suef 62511, Egypt; 5 Clinical Pharmacy Department, College of Pharmacy, King Khalid University, Abha 61421, Saudi Arabia; aribrahim@kku.edu.sa

**Keywords:** cesarean scar defect, niche, isthmocele, risk factors, complications

## Abstract

*Background and Objectives*: A cesarean scar defect (CSD), also referred to as a niche or isthmocele, is often detected incidentally during transvaginal sonography (TVS) and is typically asymptomatic. However, the exact prevalence of symptomatic niches remains unclear. This study aimed to evaluate the incidence of cesarean scar niches and identify potential risk factors in a prospectively gathered cohort of Egyptian women. *Materials and Methods*: The primary endpoint was to determine the incidence of isthmoceles after six months following a cesarean section (CS) and to investigate any associated symptoms and risk factors. The study included 420 women, divided into three groups: Group A included 140 women who had undergone their first CS, Group B included 140 women with a history of two CSs, and Group C consisted of 140 women with more than two prior CSs. *Results*: Niches were identified in 23.8% of the participants via TVS. The highest incidence was observed in women with more than two previous CSs (31.2%, 39/125), followed by those with two prior CSs (24.4%, 30/123), and the lowest was among women with one previous CS (16.3%, 22/135). Of the 91 women diagnosed with a CS niche, only 23 (25.27%) reported symptoms—most commonly postmenstrual spotting (7.7%) and dyspareunia (8.8%). *Conclusions*: The findings indicate that multiple cesarean deliveries, the uterine positioning (as assessed via TVS), a postpartum fever, breastfeeding, low post-cesarean platelet counts, and maternal anemia are contributing factors to the development of cesarean scar niches.

## 1. Introduction

A cesarean section (CS) is a surgical intervention typically performed when a vaginal delivery poses potential risks to the mother, the fetus, or both [1]. Globally, CS rates have risen significantly, reaching **25% in Europe, 42% in South America, 40% in Latin America, and up to 50% in Egypt, sparking concern about the associated long-term complications [2].

One increasingly recognized complication is the development of a cesarean scar niche (CSN), also known as a cesarean scar defect (CSD) or isthmocele. A cesarean scar niche refers to a defect resulting from poor healing of the myometrium at the site of a cesarean incision. The precise cause of niche formation remains unclear, but is believed to be multifactorial. Contributing factors may include the site of the uterine incision, uterine retroflexion [3], obstetric and gynecological conditions, and individual patient factors such as underlying medical conditions that impair wound healing [4,5,6].

CSNs can be identified in non-pregnant women using various imaging techniques such as transvaginal sonography (TVS), saline infusion sonohysterography (SHG), a 3D ultrasound, magnetic resonance imaging (MRI), or a hysteroscopy [7]. Hysterosalpingography can also be used for a diagnosis. A niche is typically defined as an anechoic area of at least 1 mm in depth (measured from base to apex) or ≥2 mm deep within the myometrium at the scar site, with or without fluid accumulation.

However, there is currently no universal diagnostic criterion, and the prevalence rates vary depending on the diagnostic method used [8]. Common diagnostic tools include two- and three-dimensional TVUS (with or without saline or gel contrast) and MRI [3].

Although many CSNs are asymptomatic, symptomatic women may present with postmenstrual spotting, dysmenorrhea, dyspareunia, chronic pelvic pain, or infertility. These symptoms are thought to result from retained menstrual blood within the niche, fibrotic tissue formation, and poor myometrial remodeling.

Though rare, serious complications such as uterine rupture or a cesarean scar ectopic pregnancy can arise from niche formation and carry significant risks [9,10]. Niche presence may also complicate gynecological procedures like intrauterine device (IUD) insertion, uterine evacuations, and embryo transfers [11].

The risk factors for CSN formation include multiple cesarean deliveries, uterine retroflexion, low post-operative hemoglobin or platelets, an infection (e.g., postpartum fever), a suboptimal surgical technique (e.g., single-layer uterine closure), or labor before the cesarean section. Additionally, maternal anemia and impaired wound healing can exacerbate the defect [12].

Management strategies for symptomatic CSNs include hormonal therapy, a hysteroscopic or laparoscopic correction, and in rare cases, surgical excision. Nonetheless, prevention remains the most effective approach, emphasizing the importance of optimized surgical techniques and the early identification of at-risk patients.

Given the high rate of CSs in Egypt and the potential reproductive consequences of CSNs, this study aimed to investigate the presence of cesarean scar niches and identify associated risk factors in a prospective cohort of Egyptian women following their first, second, or more than two cesarean sections. The findings are intended to enhance the understanding of CSN pathogenesis and support the development of preventive and management strategies to reduce its occurrence and related complications.

## 2. Materials and Methods

### 2.1. Study Population

This prospective cohort observational study was performed at the Department of Obstetrics and Gynecology, Beni-Suef University Hospital, Beni-Suef, Egypt, after obtaining ethical approval from the medical ethical committee. The recruited women provided signed informed consent before enrollment after the purpose and procedures of the study were explained.

The inclusion criteria included the following:

- Women who had undergone a cesarean section at least six months prior to the study.
- Availability for a transvaginal ultrasound (TVUS) evaluation.

The exclusion criteria included the following:

- A single-layer uterine closure technique (due to potential confounding in niche healing).
- Uterine anomalies.
- Women with diagnosed chorioamnionitis or a postpartum pelvic infection.

Accordingly, a total of 420 women were enrolled from February 2022 to January 2024, and they were categorized as follows: Group A (n = 140) women had undergone their first caesarean section (1 CS), Group B (n = 140) women group had undergone a second CS (2 CSs), and Group C (n = 140) women had undergone more than two CSs.

Data were collected for all the participants from their medical records, including the incidence, complications, and risk factors (the maternal age, the prenatal BMI, medical comorbidities, PROM, PTL, a history of a previous vaginal delivery, a VBAC or hysterotomy, the gestational age, EFW, twins, labor duration, the indication for a CS, the cervical dilatation at the time of the cesarean section, the post-cesarean hemoglobin, the platelet count or WBC count, a postpartum fever, wound infections, breastfeeding, postpartum contraceptive methods, and the uterine position).

The primary outcome of the current study was to assess the incidence of a CS niche among cesarean deliveries, and the secondary outcome was to assess the complications and risk factors that affect the development of a niche.

### 2.2. Sample Size Calculation

The primary endpoint of this study was to assess the incidence of a niche 6 months after a CS and the association of possible symptoms, with the determination of possible risk factors representing the secondary outcome of the study. Under the assumption that 30% of women present with myometrial defects after a CS [13], the cohort sample size was generated with a confidence level (CI) of 0.95; hence, the total sample size was 420 to allow for a 30% dropout rate.

### 2.3. Ultrasound Assessment

Women who fulfilled the inclusion criteria within 6 months after their cesarean delivery were examined using a 2D ultrasound (trans-abdominal and transvaginal) and saline infusion sonography (SIS) to detect the presence of a niche. Also, the women were examined in the lithotomy position with an empty bladder; the uterus was examined in a standardized way, with transvaginal ultrasonography (TVUS) performed first.

All the ultrasound examinations were conducted by a single experienced gynecologist using the same ultrasound machine to reduce inter-observer and inter-device variability.

For the diagnosis of a CS niche (defined as an anechoic defect in the anterior wall of the lower uterine segment, communicating with the endometrial cavity), the authors used a predetermined definition of a defect at least 2.0 mm deep [14]. If a cesarean scar niche was detected, the depth and width of the niche, the residual myometrial thickness (RMT) overlying the niche, and the adjacent myometrial thickness fundal to the niche were measured in the midsagittal plane. The length of the niche was measured in the transverse plane [15]. The uterine position was classified as anteverted or retroverted. In cases where >1 defect was found, the largest one was measured. Moreover, a cesarean scar niche was considered large if the ratio between the depth of the niche and the adjacent myometrial thickness was more than 50%.

### 2.4. Statistical Analysis

The data were statistically described in terms of the mean ± standard deviation (mean ± SD), frequencies (number of cases), and relative frequencies (percentages) when appropriate. A normal distribution of continuous variables of the demographic data was evaluated with the use of the Kolmogrov–Smirnov test. Comparisons of numerical variables between the study groups were facilitated using an independent-samples *t*-test (when the data showed a normal distribution) or the Mann–Whitney U test for non-normally distributed data. A chi-square (*X*^2^) test was performed for comparing categorical data in the study, and for small sample sizes, Fisher’s exact (F) test was used. Statistical significance was set at a probability value (*p*-value) ≤ 0.05. The data were analyzed using SPSS (version 22; SPSS Inc., Chicago, IL, USA).

A logistic regression model was applied to identify independent predictors of a CSN, reporting odds ratios (ORs) with 95% confidence intervals (CIs). ** Statistical significance was set at *p* ≤ 0.05.

## 3. Results

### 3.1. Incidence of Cesarean Scar Niche

A total of 420 eligible women were recruited; however, **37 participants (8.8%) were excluded from the final analysis** due to a pregnancy before 6 months (n = 2) or loss to follow-up/withdrawal (n = 35). The dropout rate varied significantly among Group A (3.6%), Group B (12.1%), and Group C (10.7%) (**p = 0.025**).

Consequently, The analysis demonstratedcases fulfilling inclusion criteria and complied to follow up for 6 months. As shown in Table 1, 91 women (23.8%) displayed a niche at the site of their CS in the TVS, with a significant difference between the three groups (*X*^2^, 7.99, *p* = 0.025).

### 3.2. Risk Factors of Cesarean Scar Niche

The levels of risk factors were compared among the three enrolled groups, as shown in Table 2. The total mean maternal age was 29.51 ± 4.7 (mean ± SD), and the maternal age differed significantly among the groups (*p* = 0.001), but showed no significant statistical difference between women who complained of a CS defect and the Other group (*p* = 0.92). The mean total prenatal BMI among the three groups was (32.78 ± 3.14); the BMI was higher in women with >2 CSs (*p* = 0.025), while no significant difference was reported between women with a CS defect and the other defect-free group. Among all the groups, a significant difference was reported between women with one CS and those with more than two CSs (*p* = 0.021). Medical comorbidities were categorized into DM (n = 3), gastrointestinal DM (n = 4), preeclampsia (n = 12), and maternal anemia (n = 88); a significant difference was only reported for the anemia group between patients who developed a CS niche and the other defect-free patients. No statistical significance was reported for the obstetric risk factors (e.g., histories of premature rupture of membranes [PROM], preterm delivery, previous vaginal delivery, VBAC, or previous hysterotomy) among the three investigated groups. Similarly, the fetal risk factors (e.g., gestational age at delivery, EFW, macrosomia, and twins) have no statistical significance difference between groups. The labor risk factors (e.g., labor duration reported in minutes, elective or emergency labor, and cervical dilatation at the time of the cesarean section) did not show statistical significance among the three investigated groups.

**Figure 1:**
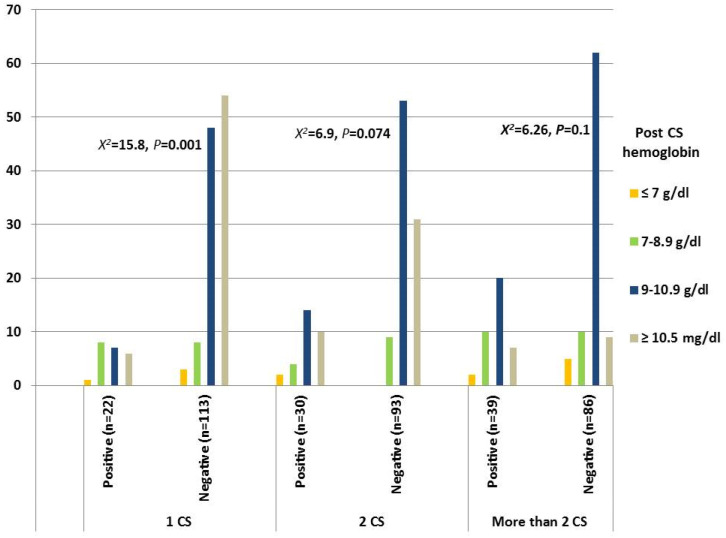
Post-cesarean hemoglobin concentration.

Regarding laboratory risk factors, the post-cesarean hemoglobin concentration dropped more in patients with a CS niche after their first delivery, with a statistically significant difference based on a chi-square test (*p* = 0.001) in patients with one previous CS. There was No statistical significant drop in hemoglobin level post CSs in patients with history oftwo previous CSs (*p* = 0.074) or more than two CSs (*p* = 0.1), as shown in Figure 1.

**Figure 2:**
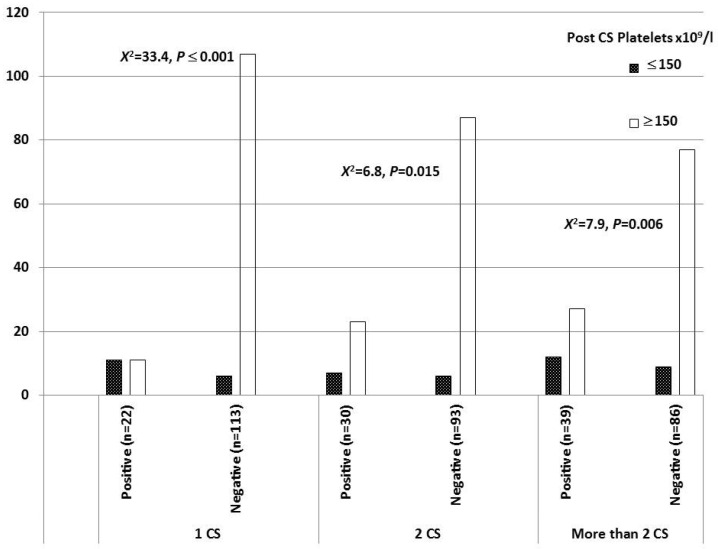
Post-cesarean platelet count. No significance was reported among the three CS groups regarding the WBC count.

The post-cesarean platelet count was lower among women who developed a CS niche (50% for those with one previous CS, 23.3% in those with two previous CSs, and 30.8% in those with more than two CSs). These women had platelet counts less than 150 × 10^9^/L and demonstrated a statistically significant difference, as shown in Figure 2.

A postpartum fever and breastfeeding showed significant statistical differences among the three CS groups, at (*X*^2^ = 14.76, *p* ≤ 0.001) and (*X*^2^ = 26.02, *p* ≤ 0.001), respectively, as shown in Table 3.

Patients who were documented with a wound infection or who used postpartum contraceptive methods reported no statistical differences among the three CS groups.

The uterine position according to a transvaginal ultrasound showed significant statistical differences among the three CS groups (*p* ≤ 0.0001), as shown in Table 4.

### 3.3. Complications

**Figure 3:**
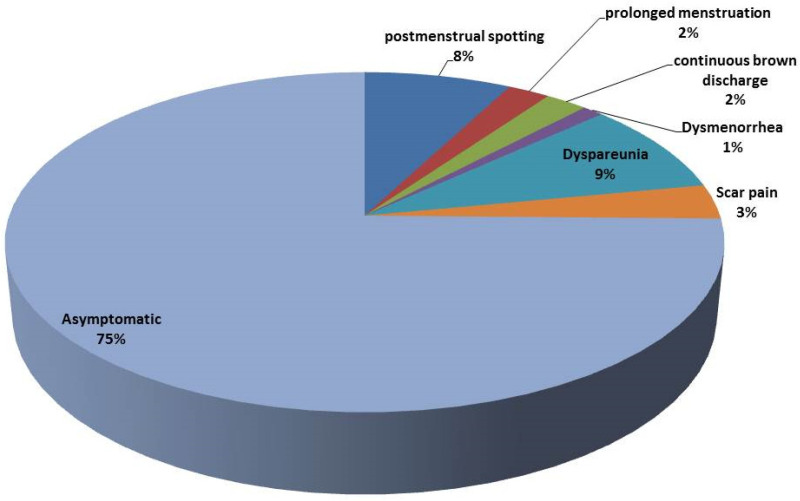
Frequencies of symptoms in symptomatic patients with a CS niche.

From 91 women with a CS niche, only 23 patients had symptoms (25.27%). The frequencies of symptoms in symptomatic patients with a CS niche are shown in Figure 3.

### 3.4. Correlation Between Factors Associated with CSs

A logistic regression model was used to investigate the factors associated with a cesarean scar niche. The model included a [CS niche] as the binary outcome and [maternal anemia, post-operative platelet count, postpartum fever, uterine position, and breastfeeding] as predictors. The overall model was statistically significant (*p* < 0.001), indicating a good fit between the data and the model.

Among the significant predictors, [maternal anemia] showed an OR of 1.66, a 95% CI of 0.668–4.125, and *p* = 0.276. [Post-operative platelets] showed an OR of 4.946, a 95% CI of 2.305–10.61, and *p* = 0.001. A [post-operative fever] showed an OR of 2.072, a 95% CI of 0.912–4.708, and *p* = 0.082. The [retroflexion position] showed an OR of 3.125, a 95% CI of 1.832–5.332, and *p* ≤ 0.001. [Breastfeeding] showed an OR of 8.8, a 95% CI of 2.803–27.623, and *p* ≤ 0.0001, as shown in Table 5.

## 4. Discussion

A cesarean scar defect (CSD), also known as a niche or isthmocele, is frequently identified incidentally during transvaginal sonography (TVS) and is often asymptomatic [16]. The prevalence of symptomatic niches remains unclear. However, with the global rise in repeat cesarean deliveries [17], the incidence of uterine scar defects is expected to increase [18]. Initially identified through hysterosalpingography, a niche typically appears on TVS as a wedge-shaped, anechoic area at the presumed site of the cesarean incision [19]. The current study aimed to prospectively evaluate the incidence of cesarean scar niches and associated risk factors.

Among the 393 women assessed, TVS detected niches in 91 cases (23.8%). The highest incidence was observed in women with more than two prior cesarean deliveries (31.2%, 39/125), followed by those with two previous CSs (24.4%, 30/123), and the lowest incidence was observed in women with one prior CS (16.3%, 22/135). These results are consistent with earlier studies [20,21] that identified multiple cesarean sections as a primary risk factor for CSD development.

Maternal age was not significantly associated with niche formation among symptomatic versus asymptomatic women, aligning with the findings of Antila et al. (2020) [22]. However, significant differences in the mean maternal age were noted across the CS groups, with women undergoing more than two CSs being older—a finding in line with Rydahl et al. (2019) [23], who linked an advanced maternal age to increased pre-pregnancy comorbidities and cesarean risk.

Obesity is associated with poor labor outcomes [24,25], such as reduced spontaneous labor, increased rates of induction, and a higher likelihood of cesarean delivery [26]. In the current study, while there was no significant BMI difference between women with or without a niche, a significant difference in the BMI was observed across the CS groups, with the highest BMI seen in women with more than two CSs. This supports prior findings linking a higher BMI to an increased cesarean risk [27].

Regarding comorbidities, only anemia showed a significant association with a CSD, consistent with previous studies [28,29]. Although a vaginal delivery is generally preferred for pregnancies complicated by preeclampsia when no contraindications exist, a cesarean delivery remains the definitive treatment [30]. This study found that a postpartum fever was significantly more common in women with multiple CSs, corroborating earlier findings that a postpartum infection and an elevated maternal temperature [31,32,33] may contribute to CSD formation [34,35]. Beyond physiological risks, a previous study [36] demonstrated that postpartum infections can negatively impact maternal mental health [37] and breastfeeding success [4].

Recent evidence also suggests that women intending to exclusively breastfeed for six months are less likely to undergo non-medically indicated cesarean deliveries [38]. In agreement, this study found breastfeeding to be associated with a reduced incidence of repeat CSs across the groups.

Interestingly, uterine retroflexion was significantly more common among women with one prior CS and a diagnosed niche (*p* < 0.001), a finding aligned with previous literature [39]. The other evaluated risk factors, such as the type of delivery (elective vs. emergency), postpartum contraceptive use [40], the WBC count, and cervical dilation, did not show significant differences among the CS groups [41]. While Pan et al. (2019) found no significant correlation between cervical dilation and niche formation [42], earlier studies [34,43,44] suggest that less cervical dilation or advanced labor at the time of the CS may increase the risk of larger niches due to incomplete myometrial approximation during uterine closure.

Most of the women with a CSD in this study were asymptomatic; only 23 of 91 (25.27%) reported symptoms such as postmenstrual spotting, dyspareunia, scar pain, prolonged menstruation, persistent brown discharge, or dysmenorrhea. The link between a CSD and abnormal uterine bleeding may be attributed to retained menstrual blood in the defect, a fibrotic obstruction, or vascular changes at the scar site [21]. Although the reported rate of postmenstrual spotting was relatively low, the association between niche formation and abnormal bleeding identified via TVS and SHG at six months postpartum is consistent with prior studies [15,22,45].

### 4.1. Clinical Implications

This study emphasizes the importance of routine CSN screening, especially in women with multiple CSs or risk factors such as uterine retroflexion or low platelet counts.

Symptom-based detection underestimates the prevalence, delaying potential interventions for subfertility or abnormal uterine bleeding.

### 4.2. Preventive Considerations

- The adoption of double-layer closure techniques.
- The optimization of the maternal hematological status pre-operatively.
- The early detection of a postpartum fever or infection.
- Postpartum follow-up using TVUS or SHG at 6 months for high-risk patients.

### 4.3. Limitations

- Data on the suture technique and intra-operative variables were not collected.
- We did not assess hormonal profiles or breastfeeding intensity.
- Ultrasound measurements, though standardized, were not confirmed by MRI.
- Confounding variables may have influenced the platelet and breastfeeding associations.

## 5. Conclusions

The current study found that multiple cesarean deliveries, the uterine position as assessed by a transvaginal ultrasound (TVUS), a postpartum fever, breastfeeding, the post-operative platelet levels, and maternal anemia are contributing factors to the development of cesarean scar defects (CSDs). These defects are detectable through a TVUS, with postmenstrual spotting or bleeding being the most commonly reported symptom. However, further research is needed to better understand the factors that influence the clinical presentation, management preferences, and treatment success. Standardized definitions and measurement criteria are essential to accurately identify the risk factors, assess the clinical significance, and evaluate the treatment outcomes.

## Figures and Tables

**Table 1:** The incidence of CS defects in relation to number of previous cesarean section.

CS Groups (n = 383)	CS Niche, n (%)		Statistic
	Positive	Negative	
**One Previous CS**	22 (16.3%)	113 (83.7%)	*X*^2^ = 7.99, *p* = 0.018
**Two Previous CSs**	30 (24.4%)	93 (75.6%)	
**More than Two CSs**	39 (31.2%)	86 (68.8%)	

**Table 2:** Mean levels (mean ± SD) and percentages (n, %) of maternal age, prenatal BMI, and medical comorbidities among the three CS groups.

Risk Factors	1 CS		2 CSs		More Than 2 CSs		Statistics
	Positive n = 22	Negative n = 113	Positive n = 30	Negative n = 93	Positive n = 39	Negative n = 86	
**Maternal age**	25.77 ± 3.77	26.65 ± 3.92	29.2 ± 3.53	29.2 ± 3.89	33.76 ± 3.12	32.74 ± 3.95	***p* = 0.001**
**Prenatal BMI**	32.45 ± 3.23	32.14 ± 3.1	32.4 ± 3.28	33.16 ± 3.13	34.36 ± 2.65	32.72 ± 3.11	***p* = 0.025**
**Medical comorbidities**							
**DM**	0 (0%)	0 (0%)	0 (0%)	1(1.1%)	1(2.6%)	1(1.2%)	*p* = 0.558
**Gastrointestinal DM**	2 (0.1%)	1(0.9%)	0 (0%)	0 (0%)	0 (0%)	1(1.2%)	*p* = 0.24
**Preeclampsia**	3 (13.6%)	4 (3.5%)	0 (0%)	2 (2.2%)	1 (2.6%)	2 (2.3%)	*p* = 0.49
**Anemia**	10 (45.5)	23 (20.4%)	8 (26.7%)	20 (21.5%)	12 (30.8%)	15 (17.4%)	***p* = 0.015**

**Table 5:** Logistic regression correlation between factors associated with CSs.

Predictors	*p*-Value	Odds Ratio	95% CI	
			Lower	Upper
**Maternal Anemia**	**0.276**	1.66	0.668	4.125
**Post-CS Platelets (Less than 150 × 10^9^/L)**	≤0.0001	4.946	2.305	10.61
**Post-Operative Fever**	0.082	2.072	0.912	4.708
**Uterus Position**	≤0.001			
**Retroflexion**	≤0.001	3.125	1.832	5.332
**Midline Position**	0.218	1.393	0.53	3.664
**Breastfeeding**	≤0.0001	8.8	2.803	27.623

**Table 3:** Percentages (n, %) of postpartum risk factors among the three CS groups.

Groups		Postpartum Fever		Breastfeeding	
		No	Yes	No	Yes
**One Previous CS**	**Positive**	15 (68.2%)	7 (31.8%)	6 (27.3%)	16 (72.7%)
	**Negative**	99 (87.6%)	14 (12.4%)	3 (2.7%)	110 (97.3%)
		*X*^2^ = 5.29, *p* = 0.047		*X*^2^ = 17.94, *p* ≤ 0.001	
**Two Previous CSs**	**Positive**	23 (76.7%)	7 (23.3%)	7 (23.3%)	23(76.7%)
	**Negative**	87 (93.5%)	6 (6.5%)	3 (3.2%)	90 (96.8%)
		*X*^2^ = 6.84, *p* = 0.015		*X*^2^ = 12.38, *p* = 0.002	
**More than Two CSs**	**Positive**	31 (79.5%)	8 (20.5%)	8 (20.5%)	31 (79.5%)
	**Negative**	80 (93.0%)	6 (7%)	2 (2.3%)	84 (97.7%)
		*X*^2^ = 4.94, *p* = 0.035		*X*^2^ = 12.1, *p* = 0.001	

**Table 4:** Percentages (n, %) of uterus position among the three CS groups.

Groups			Uterus Position			*p*-Value
			Anteflexion	Retroflexion	Midline	
**One CS**	**Positive**	10 (45.5%)		10 (45.5%)	2 (9.1%)	0.043
	**Negative**	82 (72.6%)		25 (22.1%)	6 (5.3%)	
**Two CSs**	**Positive**	17 (56.7%)		12 (40%)	1 (3.3%)	0.013
	**Negative**	72 (77.4%)		14 (15.1%)	7 (7.5%)	
**More than Two CSs**	**Positive**	24 (61.5%)		12 (30.8%)	3 (7.7%)	0.017
	**Negative**	71 (82.6%)		9 (10.5%)	6 (7.0%)	

## Data Availability

The raw data were generated at the [Obstetrics and Gynecology Department, Faculty of Medicine, Beni-Suef University]. The data supporting the findings of this study are available from the corresponding author upon request.
